# Prognostic models for identifying adults with intellectual disabilities and mealtime support needs who are at greatest risk of respiratory infection and emergency hospitalisation

**DOI:** 10.1111/jir.12376

**Published:** 2017-05-11

**Authors:** C. M. Perez, A. P. Wagner, S. L. Ball, S. R. White, I. C. H. Clare, A. J. Holland, M. Redley

**Affiliations:** ^1^ Cambridge Intellectual and Developmental Disabilities Research Group, Department of Psychiatry University of Cambridge Cambridge UK; ^2^ Health Economics Group, Norwich Medical School University of East Anglia Norwich UK; ^3^ National Institute of Health Research (NIHR) Collaborations for Leadership in Applied Health Research and Care (CLAHRC) East of England Cambridge UK; ^4^ MRC Biostatistics Unit University of Cambridge Cambridge UK

**Keywords:** dysphagia, hospital admissions, intellectual disability, physical health, respiratory illness, social care

## Abstract

**Background:**

Among adults with intellectual disabilities (ID), problems with eating, drinking and swallowing (EDS), and an associated need for mealtime support, are common, with an estimated 15% of adults known to specialist ID services requiring mealtime support. We set out to identify which adults with ID who receive mealtime support are at an increased risk of *respiratory infections* and *emergency hospitalisation related to EDS problems*.

**Method:**

An exploratory, prospective cohort study was undertaken in the East of England. At baseline, structured interviews with the caregivers of 142 adults with ID and any type of mealtime support needs were used to gather information on health and support needs over the previous 12 months. These interviews were repeated at follow‐up, 12 months later. The resulting dataset, covering a 24‐month period, was analysed with logistic regression, using model averaging to perform sensitivity analysis, and backwards step‐wise variable selection to identify the most important predictors.

**Results:**

Individuals with a *history of respiratory infections* (in the first year of study), those who had *epilepsy* and those with *caregiver‐reported difficulty swallowing* were most likely to have *respiratory infections* in the second year. Adults with *increasing mealtime support needs*, *epilepsy* and/or *full mealtime support needs* (fed mainly or entirely by a caregiver or enterally) were at increased risk of *emergency hospitalisation for EDS‐related problems*.

**Conclusions:**

Our findings highlight the importance of carefully monitoring health issues experienced by adults with ID and EDS problems, as well as their eating, drinking and swallowing skills. However, the models developed in this exploratory research require validation through future studies addressing the EDS problems commonly experienced by adults with ID and their implications for health outcomes and quality of life. Further research into the relationship between epilepsy and EDS problems would provide much‐needed insight into the complex relationship between the two areas.

## Introduction

It is well‐established that adults with intellectual disabilities (ID) experience substantial health‐related inequalities and receive healthcare inadequate to their needs (Emerson & Baines 2010). Despite legislation protecting disabled citizens' rights to equal treatment, which aims to minimise disability‐related disadvantages through ‘reasonable adjustments’ (*Equality Act 2010, s. 149*), people with ID are more likely to die from causes deemed avoidable through better quality healthcare and have a life expectancy approximately 15 years shorter than the ‘general’ population (Hollins *et al.* 1998; Glover & Ayub 2010; Heslop *et al.* 2013). Although evidence regarding rates and causes of acute hospitalisations for adults with ID is limited, best estimates indicate that, compared to the wider population without ID, a larger proportion of hospital admissions are for emergencies (Emerson *et al.* 2012), and that more of these admissions are for ‘ambulatory care sensitive conditions’ (ACSCs), which better primary care could potentially prevent (Emerson *et al.* 2012; Glover & Evison 2013). Adults with ID are particularly vulnerable to morbidity and mortality from respiratory infections, epilepsy and gastrointestinal disorders, amongst other conditions (Krahn *et al.* 2006).

Approximately 15% of adults known to specialist ID services receive some form of mealtime support as a consequence of eating, drinking and/or swallowing (EDS) problems, related to physical and behavioural issues (Ball *et al.* 2012), along with dysphagia (difficult or painful swallowing), which is thought to affect around 8% of all adults with ID (Chadwick & Jolliffe 2009). Mealtime support encompasses a diverse range of interventions: from modification of food or drink texture and prompting or pacing advice, to enteral feeding by percutaneous endoscopic gastrostomy or jejunostomy (PEG/PEJ) (Ball *et al.* 2012). Often, this support will be accompanied by formal guidelines, compiled by a speech and language therapist (SLT), dietitian and/or occupational therapist (OT), following specialist assessment (Chadwick *et al.* 2006). Recognising EDS problems, planning and implementing mealtime support require substantial collaboration between professionals [e.g. SLTs, OTs and General Practitioners (GPs)] and family carers or paid support workers, most of whom are not health professionals (Marriott & Turner 2016). Improved understanding and awareness of the predictors of adverse outcomes are crucial, as this could help professionals and caregivers alike to better identify adults with ID who are at increased risk and help prevent health problems before they occur.

Existing UK research addressing adults with ID and EDS problems has focused on characteristics of dysphagia (Chadwick & Jolliffe 2009), carer knowledge of and adherence to clinical guidelines for managing dysphagia (Chadwick *et al.* 2002; Chadwick *et al.* 2003; Chadwick *et al.* 2006; Crawford *et al.* 2007), and carers' perceptions of influences of choking risk around mealtimes, alongside their socio‐environmental management strategies (Guthrie & Stansfield 2017; Guthrie *et al.* 2015). Samuels & Chadwick (2006) investigated asphyxiation risk in adults with ID and dysphagia by considering predictors related to the oral preparatory and transfer stages of the swallow and maladaptive eating styles/environmental factors; they found that premature loss of the bolus, eating speed and cramming are significant risk predictors. See Marriott & Turner (2016) for an overview of dysphagia research findings. Previous studies addressing respiratory infections in high‐risk groups have focused primarily on elderly, non‐ID, US‐based samples: exploring predictors of aspiration pneumonia in elderly men (Langmore *et al.* 1998) and women (Langmore *et al.* 2002), and reviewing the literature on dysphagia and pneumonia (Marik & Kaplan 2003; Eisenstadt 2010). However, a recent UK‐based study, by Hibberd *et al.* (2013), investigated 26 ‘influencing factors’ for aspiration pneumonia in a group of 687 patients referred to SLTs for suspected dysphagia. This included small numbers of adults with ID (*N* = 25) or head/neck cancer, alongside a majority of ‘other’ adults (mostly acute hospital inpatients). Thirteen factors were statistically significant predictors of aspiration pneumonia, the most important including the following: receiving ‘mixed’ oral and tube feeding; having ‘poor mobility’; being older, ‘dependent’ for feeding, and/or exclusively orally fed; and having dysphagia and/or a larger ‘number of medical conditions’; amongst other factors (Hibberd *et al.* 2013). However, as with the above studies of elderly individuals, Hibberd *et al.*'s study population had a mean age of 72.9 years (over 20 years older, on average, than our study population), and their analysis focused on inpatients, as there were too few individuals with ID to model separately.

Age is an important focus in most EDS‐related research, which emphasises changes to the physiological swallowing process that accompany ageing, as well as the associations between older age and other conditions that can themselves cause EDS problems (e.g. stroke and dementia). Beyond emerging as an important predictor of aspiration pneumonia in Hibberd *et al.*'s (2013) study, in the US, Sheppard (2002) highlights age‐related deterioration of feeding ability in people with severe/profound ID (which occurred in three‐quarters of that study's participants), along with the fact that these age‐related changes may emerge relatively early, starting in the 30s. Several Australian studies of people with cerebral palsy (CP) have found that their EDS abilities, and mealtime support needs, change with age; findings, which given the increased longevity of adults with ID, suggest a need for further research (Balandin 2002; Balandin *et al.* 2009). These studies highlight the need to monitor both individuals who have always required mealtime support (as their needs may increase with age), and those who have historically been EDS problem‐free, but develop issues as a result of other chronic conditions, or simply because of age‐related deterioration in swallowing (Chadwick & Jolliffe 2009). Older people (with ID and without) are more likely to experience both respiratory infections and hospitalisation (Balandin 2002); therefore, we consider age an important predictor.

Building on existing research, we sought to identify variables that best predict *respiratory infections* and *emergency hospitalisations related to EDS problems* in a sample of adults with ID and mealtime support needs, by developing suitable prognostic regression modelling (Moons *et al.* 2009). Such modelling typically consists of three stages: model development, validation and, finally, impact studies. We focus on model development. Given our sample size, we are unable to formally validate the models; however, the work presented is an important first step to address these EDS problems in this vulnerable and difficult‐to‐recruit population. Finally, within the constraints of the dataset, we use statistical model averaging and sensitivity analysis to explore robustness of the developed models and to assess the impact of untestable assumptions, such as the form of the age variable included in the models, showing that the results are not sensitive to model‐structural mis‐specification.

We aim to fill the gap between what is known about predictors of respiratory infections in elderly populations and more general research on ill‐health and hospitalisation among adults with ID. To our knowledge, this is the first study to explore predictors of respiratory infections and emergency hospitalisations in adults with ID who require mealtime support.

## Methods

### Study setting and data collection

This research draws on data collected in an observational study carried out in two counties in the East of England. Inclusion criteria were simply that potential participants were adults (≥18 years old) with an ID, who received mealtime support for any kind of problem with eating and/or drinking. Recruitment therefore focused on need for and the receipt of support, rather than on underlying clinical diagnoses (e.g. dysphagia). Eligible individuals were identified from the population known to the local specialist Community Learning Disability Teams, over a 12‐month period, beginning in July 2008 in Cambridgeshire and December 2008 in Essex (excluding southeast Essex). All identified individuals were invited to participate. Consent or favourable advice was sought for interviews with caregivers (family carers, paid support workers or care home managers) and healthcare practitioners involved in the provision of mealtime support, and for access to health notes. Participants were interviewed at baseline in face‐to‐face interviews, using a structured *pro forma* (with data collected between January and September 2009), and at follow‐up 12 months later by telephone (between January and September 2010). All interviews were conducted by research team members.

The baseline interview gathered cross‐sectional and retrospectively reported information concerning the year prior to interview (referred to as ‘year one’). This resulted in variables that collected information across several areas: *sociodemographic characteristics* – age, gender, living arrangements; *disability‐related characteristics* – severity of ID, CP diagnosis, Down syndrome diagnosis, presence of physical disability, level of mobility; *mealtime support needs* – difficulty self‐feeding, mealtime support level, whether mealtime support needs had increased prior to baseline interview (‘stability’); *indicators of dysphagia* – dysphagia diagnosis, carer‐reported swallowing problems, clinical features of dysphagia (coughing, choking, shortness of breath or ‘gurgly’ voice around mealtimes); and *other conditions or illnesses* – suspected or diagnosed dementia, epilepsy, history of respiratory infection (in the first year of the study); see Table 1 and Perez *et al*. (2015), for further details. Mealtime support level was characterised as: ‘minimal or moderate’ support, including assistance with modifying food/drink texture, prompting and pacing advice, correct positioning and the use of specialist equipment, or ‘full’ support, for individuals fed entirely by someone else or enterally via PEG/PEJ tube. With respect to the stability of mealtime support needs, ‘stable’ needs refer to individuals who had always required support and whose EDS skills had not deteriorated over time, whereas ‘increasing’ needs included people whose caregiver reported a deterioration in their EDS skills, resulting in greater support needs pre‐baseline. Except age, all variables are categorical; we use dichotomisations of all categorical variables (see Tables 4, 5).

**Table 1:** Baseline characteristics of all participants by county of residence (*N* = 127, excluding the eight people who died and seven lost to follow‐up). Bolded text highlights *P*‐values ≤ 0.05

Baseline characteristics		County of residence (*N* = 127), *n* (column %) a		Unadjusted OR (95% CI) b	*P*‐value c
		Cambridgeshire (*N* = 62)	Essex (*N* = 65)		
*Socio‐demographic characteristics*	*Age at baseline (in years):*				
	Mean (standard deviation)	42.8 (16.3)	50.3 (18.3)	—	**0.023**
	Median (interquartile range)	45.5 (25.8–56.3)	49.75 (35.5–66.0)		
	*Gender:*				
	Male	28 (45%)	42 (65%)	0.5 (0.2–0.9)	**0.033**
	Female	34 (55%)	23 (35%)		
	*Living arrangements*:				
	Residential care	57 (92%)	52 (80%)	2.9 (1.0–8.5)	0.074
	Own/family home	5 (8%)	13 (20%)		
*Disability‐related characteristics*	*Severity of ID (n = 126):*				
	Mild/moderate	17 (27%)	25 (39%)	0.6 (0.3–1.2)	0.189
	Severe/profound	45 (73%)	39 (61%)		
	*Has cerebral palsy (CP)*	*19 (31%)*	*19 (29%)*	*0.9 (0.4–2.0)*	*1.000*
	*Has Down's syndrome (DS)*	*13 (21%)*	*5 (8%)*	*0.3 (0.1–0.9)*	***0.042***
	*Has a physical disability*	*49 (79%)*	*53 (82%)*	*1.2 (0.5–2.8)*	*0.824*
	*Extent of mobility:*				
	Fully mobile	17 (27%)	19 (29)	0.9 (0.4–2.0)	0.846
	Limited/no mobility	45 (73%)	46 (71%)		
	*Has difficulty self‐feeding*	23 (37%)	32 (49%)	1.6 (0.8–3.3)	0.210
*Mealtime support needs*	*Level of mealtime support:*				
	Minimal/moderate	46 (74%)	38 (59%)	2.0 (1.0–4.3)	0.091
	Full (oral or enteral)	16 (26%)	27 (42%)		
	*Stability of mealtime support (n = 126):*				
	Stable needs	31 (50%)	32 (50%)	1.0 (0.5–2.0)	1.000
	Increasing needs	31 (50%)	32 (50%)		
*Indicators of dysphagia*	*Has diagnosed dysphagia (n = 125):*				
	No	44 (71%)	36 (57%)	1.8 (0.9–3.8)	0.137
	Yes	18 (29%)	27 (43%)		
	*Has swallowing problems*	20 (32%)	38 (59%)	3.0 (1.4–6.1)	**0.004**
	*Has any (≥1) clinical features of dysphagia in year 1*	32 (52%)	41 (63%)	1.6 (0.8–3.3)	0.212
*Other illness/disability*	*Has dementia (diagnosed or suspected)*	7 (11%)	3 (5%)	0.4 (0.1–1.5)	0.199
	*Has epilepsy*	25 (40%)	17 (26%)	0.5 (0.2–1.1)	0.131
	*Has a history of respiratory infections (in year 1)*	20 (32%)	27 (42%)	1.5 (0.7–3.1)	0.358

a Percentages may not sum to 100% due to rounding.

b OR, odds ratio; CI, confidence interval. An OR ≤ 1 indicates that the second level of a characteristic is less likely in Essex, when compared with Cambridgeshire (i.e. being female is less likely in Essex), whereas an OR ≥ 1 indicates that it is more likely (i.e. a participant living in their own/family home is more likely in Essex). Additionally, for those characteristics where no comparator category is shown, an implicit comparison is being made with the negative—e.g. ‘Has cerebral palsy (CP)’ is compared to ‘Does not have CP’.

c All of the *P*‐values presented are from Fisher's exact test, except for *Age at baseline*, where the Mann–Whitney *U* test is used.

At follow‐up, we collected information about the intervening year (‘year two’), with respect to the following: primary and secondary healthcare use; each participant's vital status over the second year and other information (e.g. mealtime support needs). Based on information from follow‐up, two outcome variables were identified: *respiratory infection* (respiratory infections resulting in GP contact or emergency hospitalisation) and *emergency hospitalisation related to EDS problems*. Eating, drinking and swallowing‐related problems were defined as any condition or illness that could result from or affect a participant's ability to eat and drink – e.g. malnutrition, dehydration, weight loss, gastro‐oesophageal reflux, regurgitation, etc. For further details about data collection, ethical considerations and the particular EDS difficulties faced by the participants, see Ball *et al*. (2012). Descriptive statistics and more detailed information about all variables, alongside other study information, can be found in Perez *et al*. (2015). The univariate analyses presented in Tables 1 and 2 were completed in IBM SPSS v.19 (IBM Corp. Released 2010). All other analyses conducted using R (R Core Team 2014).

**Table 2:** Unadjusted (crude) associations between baseline characteristics (covariates) and outcomes: (1) *respiratory infections* and (2) *emergency hospitalisations related to EDS* in the second year. Bolded text highlights *P*‐values ≤ 0.05

Baseline characteristics (covariates)			Respiratory infections in the second year: ≥1 (*N* = 122)				Emergency hospitalisations for EDS in the second year: ≥1 (*N* = 127)			
			*n* (column %) a				*n* (column %) a			
			None (*n* = 88)	One or more (*n* = 34)	Unadjusted OR (95% CI) b	*P*‐value c	None (*n* = 109)	One or more (*n* = 18)	Unadjusted OR (95% CI) b	*P*‐value c
Socio‐demographic characteristics	*Age in years at baseline*	Mean (SD)	45.8 (16.5)	52.5 (18.3)	—	**0.050**	45.8 (17.8)	51.9 (16.5)	—	0.121
		Median (IQR)	45.0 (28.0)	54.0 (25.0)			45.0 (31.0)	52.5 (19.0)		
	*Gender*	Male	45 (51%)	22 (65%)	0.6 (0.3–1.3)	0.224	57 (52%)	13 (72%)	0.4 (0.1–1.3)	0.132
		Female	43 (49%)	12 (35%)			52 (8%)	05 (28%)		
	*County*	Cambridgeshire	45 (51%)	12 (35%)	1.9 (0.8–4.3)	0.157	53 (49%)	09 (50%)	0.9 (0.3–2.6)	1.000
		Essex	43 (49%)	22 (65%)			56 (51%)	09 (50%)		
	*Living arrangements*	Residential care	75 (85%)	29 (85%)	1.0 (0.3–3.0)	1.000	92 (84%)	17 (94%)	0.3 (0.4–2.6)	0.466
		Own/family home	13 (15%)	5 (15%)			17 (16%)	1 (6%)		
Disability‐related characteristics	*Severity of ID* d	Mild/moderate	27 (31%)	13 (38%)	0.7 (0.3–1.7)	0.520	34 (32%)	08 (44%)	0.6 (0.2–1.6)	0.292
		Severe/profound	60 (69%)	21 (62%)			74 (69%)	10 (56%)		
	*Has cerebral palsy (CP)*		21 (24%)	12 (35%)	1.7 (0.7–4.1)	0.256	31 (28%)	07 (39%)	1.6 (0.6–4.5)	0.409
	*Has Down's syndrome (DS)*		12 (14%)	6 (18%)	1.4 (0.5–4.0)	0.578	14 (13%)	04 (22%)	2.0 (0.6–6.7)	0.285
	*Has a physical disability*		66 (75%)	31 (91%)	3.4 (1.0–12.4)	**0.050**	87 (80%)	15 (83%)	1.3 (0.3–4.8)	1.000
	*Extent of mobility*	Fully mobile	32 (91%)	3 (9%)	5.9 (1.7–20.9)	**0.003**	34 (31%)	02 (11%)	3.6 (0.8–16.7)	0.096
		Limited/no mobility	56 (64%)	31 (91%)			75 (69%)	16 (89%)		
	*Has difficulty self‐feeding*		30 (34%)	22 (65%)	3.5 (1.5–8.1)	**0.004**	43 (39%)	12 (67%)	3.1 (1.1–8.8)	**0.040**
Mealtime support needs	*Level of mealtime support*	Minimal/moderate	64 (73%)	16 (47%)	3.0 (1.3–6.8)	**0.011**	78 (72%)	06 (33%)	5.0 (1.7–14.6)	**0.003**
		Full (oral or enteral)	24 (27%)	18 (53%)			31 (28%)	12 (67%)		
	*Mealtime support stability* d	Stable needs	53 (61%)	5 (15%)	9.0 (3.2–25.6)	**<0.001**	061 (57%)	02 (11%)	10.4 (2.3–47.4)	**0.001**
		Increasing needs	34 (39%)	29 (85%)			047 (44%)	16 (89%)		
Dysphagia	*Diagnosed dysphagia* d	No	61 (71%)	15 (44%)	3.1 (1.4–7.0)	**0.011**	074 (69%)	06 (33%)	4.5 (1.6–13.0)	**0.007**
		Yes	25 (29%)	19 (56%)			033 (31%)	12 (67%)		
	*Has swallowing problems*		33 (38%)	24 (71%)	4.0 (1.7–9.4)	**0.001**	046 (42%)	12 (67%)	2.7 (0.96–7.8)	0.073
	*Dysphagia features, year 1*		45 (51%)	26 (77%)	3.1 (1.3–7.6)	**0.014**	061 (56%)	12 (67%)	1.6 (0.6–4.5)	0.450
Other	*Dementia (suspected/diag.)*		4 (5%)	06 (18%)	4.5 (1.2–17.1)	**0.028**	06 (6%)	04 (22%)	4.9 (1.2–19.6)	**0.035**
	*Has epilepsy*		21 (24%)	19 (56%)	4.0 (1.8–9.3)	**0.001**	030 (28%)	12 (67%)	5.3 (1.8–15.3)	**0.002**
	*Respiratory infect. in year 1*		20 (44%)	26 (57%)	11.0 (4.3–28.2)	**<0.001**	34 (72%)	13 (28%)	5.7 (1.9–17.4)	**0.001**

a The number of participants (*n* = 127) excludes individuals who died during the study period (*n* = 8) and those who could not be followed up (*n* = 7). For *respiratory infections*, it additionally excludes (*n* = 5) participants who provided no information regarding GP visits and had no hospitalisations for respiratory infections. Percentages may not sum to 100% due to rounding.

b OR, odds ratio; CI, confidence interval. An OR ≤ 1 indicates that the risk in the second category level is lower (i.e. for females on *respiratory infections*), whereas an OR ≥ 1 indicates higher risk. Where no comparator category is shown, a comparison is being made with the negative—e.g. ‘Has cerebral palsy (CP)’ is compared to ‘does not have CP’.

c All of the *P*‐values presented are from Fisher's exact test, except for *Age at baseline*, which is drawn from the Mann–Whitney *U* test.

d Missing information: ‘Severity of ID’ and ‘Mealtime support stability’ not known for one individual, and ‘Diagnosed dysphagia’ not known for two individuals. Participants with missing information on these variables had *no respiratory infections* and *no hospitalisations*.

### Statistical analysis

The analysis begins with univariate comparisons between participants using Fisher's exact test and Mann–Whitney's *U* test to explore differences in *age*, the only continuous variable. We explore if there are differences in covariates between counties (Table 1). Then, between those who experienced each outcome in the second year and those who did not (Table 2).

Next, the analysis uses prognostic modelling, which aims to find the best set of predictors for a given outcome. There is no consensus on how this should be done (Royston *et al.* 2009). We adopted the common approach of backward elimination, using the Akaike Information Criterion (AIC), to determine which variables to include. The AIC provides a measure of relative model fit amongst a set of possible models, accounting for overfitting by penalising larger models (Altman *et al.* 2009). When choosing between models using AIC, one chooses the model with the lowest AIC as this is taken to indicate this model has the ‘best’ set of variables for fitting to the given data, excluding additional variables that do not sufficiently contribute to improving the model. We also had to consider how age – the only continuous variable – should be included and chose to model it with fractional polynomials. However, the inclusion of fractional polynomials creates a problem for applying backward elimination in the standard fashion: a reduction of statistical power to learn the shape and parameters, described in *Appendix S1*. Therefore, we adopted a slightly augmented approach, focusing on inference for parameter values, and allowing shape to be assessed by a sensitivity analysis (using model‐averaging). Our small sample does not support validation of the final prognostic models, but we explore their robustness by (1) comparing with a model with all covariates and a linear form of age and (2) model averaging (see Tables 4, 5). Finally, we report key information from the prognostic models.

#### Final covariate selection approach for the prognostic models

We used a two‐stage model fitting approach for each of the two outcomes. Based on the literature reviewed, we believed that age was an important *a priori* predictor and required that any final model included it; however, we were not sure of the best form of age. Thus, in the first stage of the model fitting process, we compared 13 models which included all predictors considered above, but differed in the included form of age: they either had a single age term (one of the seven fractional polynomial forms), or two age terms (linear age and one of the other six fractional polynomial forms). From these, we picked the model with the lowest AIC and used the corresponding form of age in all subsequent models. Then, in the second stage, we proceeded with backward elimination as usual, but included the constraint that the included form of age could not be eliminated. The resulting model is the one that we report as our prognostic model.

#### Exploring robustness of final prognostic models

All prognostic models should be validated (Royston *et al.* 2009). Given our small sample, it is unfeasible to adopt the common approach of splitting the data into separate training and validation sets; thus, our models are not validated. However, we investigated the robustness of the final prognostic models by combining several approaches. Within each outcome, we compared the final prognostic model with one that included all predictors and linear age; similar parameters and *P*‐values between models provide evidence for robustness. When comparing the different forms of age in the first stage of the model fitting process, we found that there was little difference between the models; AIC values were very similar (Table 3). Given this, we computed model‐average parameter estimates using AICcmodavg (see Mazerolle 2015), for the non‐age‐related predictors in these models (with the contributions from each model weighted by the AIC of the model) (Claeskens & Hjort 2008). These averaged parameter values are then compared with parameter values from the prognostic model; where they are similar, there is evidence for the robustness of the prognostic model.

**Table 3:** Comparison of the Akaike Information Criterion (AIC) of the regression models for respiratory infection or hospitalisation. Lower AIC values indicate a better fitting model (the italicised values indicate the lowest AIC for the different model outcomes). These models include all the covariates described in the methods but differ in the form of age included

Form of age^a^	Degrees of freedom	Akaike Information Criterion (AIC) Values	
		Respiratory infection	Emergency hospitalisation related to EDS
Age	18	114.110	97.731
Age^−2^	18	115.759	97.770
Age^−1^	18	115.504	97.785
Age^−0.5^	18	115.233	97.785
Log(Age)	18	114.884	97.775
Age^0.5^	18	114.495	97.755
Age^2^	18	*113.490*	*97.697*
Age + Age^−2^	19	113.744	99.493
Age + Age^−1^	19	113.915	99.469
Age + Age^−0.5^	19	114.016	99.473
Age + Log(Age)	19	114.126	99.489
Age + Age^0.5^	19	114.240	99.515
Age + Age^2^	19	114.568	99.628

a The different forms of age are standardised to a mean of zero and unit variance.

#### Reporting the final prognostic models

For each prognostic model, we report the area under the receiver operating characteristic curve (AUROCC) as an indicator of the model's predictive ability. Area under the receiver operating characteristic curves are interpreted in line with Hosmer & Lemeshow (2000). Bootstrapping was used to produce 95% confidence intervals (CIs) around the AUROCCs. We report sensitivity and specificity plots for the prognostic models (Figs S1 and S2), with bootstrapping used to produce confidence bands around the sensitivity/specificity lines. All bootstraps are based on *n* = 5000 replicates. Calculation of AUROCCs, associated bootstrapping and sensitivity/specificity plots were calculated using the pROC package (Robin *et al.* 2011). Parameters from the models are reported as odds ratios (ORs).

## Results

### Study participants and descriptive statistics

As described in Perez *et al.* (2015), all 726 individuals identified in the prevalence study (327 in Cambridge and 399 in Essex) were invited to participate. Participation agreement (either consent or favourable advice) was given by 142 (20%). We required follow‐up data to determine if someone had experienced a respiratory infection or an emergency hospital visit within the second year. Thus, we excluded individuals who were lost to follow‐up and known to be alive (*n* = 4); lost to follow‐up and had unknown vital status (*n* = 3); and deceased by follow‐up (*n* = 8). Therefore, the regression models draw on data from 127 individuals, with a mean age of 46.6 years.

Basic descriptive statistics for variables used as covariates in the regression models are reported in Perez *et al.* (2015), comparisons between counties on these covariates are given in Table 1 and unadjusted (crude) associations between the covariates and both outcomes are given in Table 2. In the models that follow, we exclude *county* from consideration, because Table 1 suggests that county differences are primarily driven by individual characteristics. Further, the sample size would not support investigation of interactions between county and these characteristics.

### Respiratory infection

#### Linear‐age model

Eight of the 127 participants had missing data, reducing the sample size to 119 [missing data: *respiratory infection* (5); *severity of ID* and *diagnosed dysphagia* (1); *stability of mealtime support* (1); *diagnosed dysphagia* (1)]. Of these, around 29% (34/119) had a respiratory infection within the study's second year. The fit of the linear‐age model is shown in Table 4 (first group of columns). The strongest and only significant (at the 5% level) predictors of *respiratory infection* in the second year were *epilepsy* (*P* = 0.014) and *respiratory infection in the first year* (*P* = 0.002): having epilepsy or a history of respiratory infections was linked with a greater risk of an illness. While not significant, both *increasing mealtime support* (OR = 3.20; *P* = 0.084) and *swallowing problems* (OR = 4.12; *P* = 0.072) gave some evidence of a medium effect on increasing the likelihood of an illness. Similarly, living in their own or the family home had the third largest (OR = 5.69), although not significant (*P* = 0.126), effect associated with increasing the chance of an illness.

**Table 4:** Fits of the regression models relating to a respiratory infection (resulting in GP contact or an emergency hospital admission) within the last year. Hyphens indicate that a particular covariate has been excluded from a model; cells containing ‘MA’ emphasise that the columns in which they occur are model averaged (MA) estimates from across the different forms of the age relationship (see Table 3). Bolded text highlights statistically significant associations (*P*‐values ≤ 0.05)

Respiratory infection (GP contact or emergency hospitalisation) models (*N* = 119)		Linear‐age model				Model averaging across age forms from Table 3			Resulting backwards stepwise model starting from best AIC model in Table 3			
			95% CI a				95% CI			95% CI		
Covariate	Reference category	OR b	Lower	Upper	*P*‐value	OR	Lower	Upper	OR	Lower	Upper	*P*‐value
*Intercept*	—	0.00	0.00	0.04	—	0.00	0.00	0.06	0.01	0.00	0.03	—
*Age (linear form)* c	—	1.64	0.78	3.74	0.208	MA	MA	MA	—	—	—	—
*Age* ^*2*^ *(quadratic form)*	—	—	—	—	—	MA	MA	MA	1.76	0.98	3.34	0.067
*Gender: Female*	Male	0.65	0.17	2.37	0.514	0.65	0.18	2.39	—	—	—	—
*Living arrangements: Own/family home*	Residential care	5.69	0.65	59.50	0.126	4.77	0.50	45.60	5.25	0.74	40.44	0.099
*Severity of ID: Severe/profound*	Mild/moderate	0.69	0.16	2.92	0.610	0.68	0.16	2.88	—	—	—	—
*Cerebral Palsy: Yes*	No	0.91	0.20	4.25	0.907	1.05	0.21	5.32	—	—	—	—
*Down syndrome: Yes*	No	2.59	0.19	27.83	0.437	2.88	0.25	32.90	—	—	—	—
*Physical disability: Yes*	No	1.82	0.18	22.54	0.616	1.80	0.17	18.93	—	—	—	—
*Extent of mobility: Limited/no mobility*	Mobile	1.02	0.11	11.55	0.988	0.94	0.09	9.91	—	—	—	—
*Difficulty self‐feeding: Yes*	No	1.00	0.15	5.68	0.998	1.09	0.18	6.80	—	—	—	—
*Level of mealtime support: full (oral/enteral)*	Minimal/moderate	1.09	0.15	6.95	0.929	0.81	0.10	6.42	—	—	—	—
*Stability of mealtime support: increasing needs*	Stable needs	3.20	0.88	13.00	0.084	3.18	0.84	12.10	3.40	1.01	12.84	0.055
*Diagnosed dysphagia: Yes*	No	0.85	0.15	4.68	0.853	0.91	0.17	4.96	—	—	—	—
*Swallowing problems: Yes*	No	4.12	0.92	20.94	0.072	4.31	0.90	20.80	4.66	1.47	16.89	**0.012**
*Clinical features of dysphagia in year 1: Yes, ≥1*	No (zero features)	2.01	0.51	8.78	0.329	2.16	0.51	9.14	—	—	—	—
*Dementia (diagnosed or suspected): Yes*	No	3.83	0.27	38.14	0.264	4.34	0.40	47.30	4.91	0.79	35.08	0.096
*Epilepsy: Yes*	No	6.46	1.58	32.19	**0.014**	6.91	1.53	31.18	6.44	1.95	24.55	**0.003**
*History of respiratory infections (in year 1): Yes*	No	10.59	2.59	55.03	**0.002**	10.44	2.32	47.08	10.66	3.45	38.95	**<0.001**

a Odds ratio (OR) forms, with 95% confidence intervals (CI), for the coefficients are reported.

b An OR > 1 indicates a greater risk of infection.

c Age forms standardised to a mean of zero and unit variance.

#### Best form of age for the prognostic model

Thirteen models are fitted which include all the covariates of the linear‐age model but with different forms of age. The AIC of these models are reported in Table 3. The best (lowest) AIC is found with the model that only includes a quadratic form of age (AIC = 113.490); however, the range of AICs is very small, suggesting little difference between the models.

#### Selection of covariates for the prognostic model

We apply a (backwards) step‐wise procedure for determining the most parsimonious model for the prediction of a respiratory infection. The starting model for this procedure has all the covariates of the linear‐age model, but a quadratic form of age (the best form of age from Table 3); age is constrained to remain in the model. The model returned by this procedure is given in Table 3 (last group of columns). The remaining covariates have similar coefficients and *P*‐values to the linear‐age model, although the *P*‐values of the prognostic model are generally smaller. This suggests that the resulting model is reasonably robust. The positive coefficient of quadratic age indicates that there is a u‐shaped relationship reaching a minimum around 50: younger and older ages are associated with an increased risk of respiratory infection.

#### Exploring robustness of the prognostic model and summary figures

Given little difference between the models with the different forms of age (Table 3), comparing the parameters of the final prognostic model with the model averaged estimates is informative (Table 4, middle group of columns, averaged across all 11 models reported in Table 3). Where the parameters are in broad agreement, it suggests that using a quadratic form of age does not lead to markedly unusual results (compared to using other forms of age). In all cases, the model averaged estimates are quite similar to the prognostic model parameters, providing further evidence of robustness.

The prognostic model has an AUROCC of 0.91 (5000 bootstrap replicates give a 95% CI: 0.84, 0.97), corresponding to the categories of ‘excellent’ (0.8–0.9) and ‘outstanding’ (above 0.9) (Hosmer & Lemeshow 2000). Figure S1 plots the sensitivity and specificity of this model.

### Emergency hospitalisation related to eating, drinking and swallowing

#### Linear‐age model

Three of the 127 followed up had missing data, reducing the sample size to 124 [missing data: *severity of ID* and *diagnosed dysphagia* (1); *stability of mealtime support* (1); *diagnosed dysphagia* (1)]. Of these, around 15% (18/124) had an EDS‐related emergency hospitalisation within the study's second year. The fit of the linear‐age model is shown in Table 5 (first group of columns). The strongest and only significant (at the 5% level) predictors of hospitalisation were *stability of mealtime support*, where need for increasing support was associated with an increased risk of hospitalisation (OR = 7.41, *P* = 0.039), and having *epilepsy*, which also increased the risk of hospitalisation (OR = 6.30, *P* = 0.022). There was some evidence (*P* = 0.091) of a relatively large effect (OR = 5.33) of the need for *full mealtime support* increasing the likelihood of hospitalisation. Notable, but non‐significant effects include *physical disability*, where the presence of a reported physical disability *reduced* the risk of a hospitalisation (OR = 0.29, *P* = 0.344), while *diagnosed dysphagia* increased it (OR = 3.33, *P* = 0.204).

**Table 5:** Fit of the regression models relating to an emergency hospital attendance or admission for an eating or drinking issue within the last year. Hyphens indicate that a particular covariate has been excluded from a model; cells containing MA emphasise that the columns in which they occur are model averaged (MA) estimates from across the different forms of the age relationship (see Table 1). Bolded text highlights statistically significant associations (*P*‐values ≤ 0.05)

Emergency hospital visit(s) for eating and drinking difficulties (*N* = 124)		Linear‐age model				Model averaging across age forms from Table 3			Resulting backwards stepwise model starting from best AIC model in Table 3			
			95% CI a				95% CI			95% CI		
Covariate	Reference category	OR b	Lower	Upper	*P*‐value	OR	Lower	Upper	OR	Lower	Upper	*P*‐value
Intercept	—	0.04	0.00	0.30	—	0.04	0.00	0.39	0.02	0.00	0.11	—
Age (linear form) c	—	1.12	0.44	2.96	0.815	MA	MA	MA	—	—	—	—
Age^2^ (quadratic form)	—	—	—	—	—	MA	MA	MA	1.46	0.76	2.79	0.248
Gender: female	Male	0.52	0.10	2.30	0.398	0.51	0.11	2.33	—	—	—	—
Living arrangements: Own/family home	Residential care	0.38	0.01	4.06	0.464	0.35	0.02	4.93	—	—	—	—
Severity of ID: Severe/profound	Mild/moderate	0.41	0.07	2.15	0.301	0.40	0.07	2.15	—	—	—	—
Cerebral Palsy: Yes	No	1.00	0.15	6.55	0.997	1.01	0.16	6.44	—	—	—	—
Down syndrome: Yes	No	2.01	0.04	70.82	0.712	2.04	0.05	83.40	—	—	—	—
Physical disability: Yes	No	0.29	0.01	3.39	0.344	0.29	0.02	3.88	0.14	0.02	1.02	0.054
Mobility: Limited/no mobility	Mobile	0.68	0.03	16.85	0.799	0.67	0.03	13.09	—	—	—	—
Difficulty getting food to mouth: Yes	No	0.65	0.07	4.71	0.675	0.64	0.08	4.81	—	—	—	—
Level of mealtime support: full (oral/enteral)	Minimal/moderate	5.33	0.79	41.03	0.091	5.07	0.69	37.03	5.63	1.31	29.27	**0.026**
Stability of mealtime support: increasing needs	Stable needs	7.41	1.33	68.91	**0.039**	7.69	1.10	53.78	8.68	1.95	62.63	**0.011**
Dysphagia diagnosis: Yes	No	3.33	0.54	24.37	0.204	3.40	0.53	21.94	3.42	0.86	14.89	0.086
Swallowing problems: Yes	No	1.44	0.25	7.76	0.669	1.45	0.27	7.68	—	—	—	—
Dysphagia features: Yes, ≥1	No (zero features)	0.39	0.06	2.15	0.293	0.39	0.07	2.28	—	—	—	—
Dementia (diagnosed or suspected): Yes	No	1.24	0.02	33.08	0.910	1.29	0.03	49.99	—	—	—	—
Epilepsy diagnosis: Yes	No	6.30	1.40	34.77	**0.022**	6.44	1.33	31.29	5.72	1.52	25.38	**0.014**
History of respiratory infection (in year 1): Yes	No	2.77	0.52	17.64	0.245	2.80	0.50	15.57	—	—	—	—

a Odds ratio (OR) forms, with 95% confidence intervals (CI), for the coefficients are reported.

b An OR > 1 indicates a greater risk of emergency hospitalisation.

c Age forms standardised to a mean of zero and unit variance.

#### Best form of age for the prognostic model

A series of 13 models are fitted, each with a different age form. Akaike Information Criterion values for these models are shown in Table 3. There is little difference in AIC values between models, but those with a one parameter age form are preferred. The model that only includes a quadratic age form has the best AIC.

#### Selection of covariates for the prognostic model

The model returned by the step‐wise procedure is given in Table 5 (last group of columns). Broadly, the remaining covariates have similar coefficients and *P*‐values to the linear‐age model, suggesting that the resulting model is reasonably robust. However, the coefficient of *Physical disability* has changed notably (0.29 to 0.14, a fairly big difference on the OR scale). The coefficient of quadratic *age* is again positive, meaning that a greater risk of hospitalisation is associated with younger and older individuals, with a minimum around age 50.

#### Exploring robustness of the prognostic model and summary figures

There are small differences between the model averaged parameters (Table 5, middle group of columns, averaged across the 11 models reported in Table 3) and the prognostic model parameters, suggesting robustness in the prognostic model, and the prognostic model has an AUROCC of 0.89 (5000 bootstrap replicates gives a 95% CI: 0.80, 0.95). Figure S2 plots the sensitivity and specificity of this model.

## Discussion

### Summary

We set out to explore predictors of two adverse outcomes: *respiratory infections* and *emergency hospitalisation* in a group of adults with ID requiring mealtime support for EDS‐related difficulties, such as dysphagia. There is limited research on adults with ID and EDS problems, and to our knowledge, this is the first study to explore predictors of respiratory infections and emergency hospitalisation related to EDS among adults with ID and mealtime support needs.

We found that the most important risk factors for *respiratory infections* were having *a history of respiratory infection (in the previous year)*, *epilepsy* and *caregiver‐reported swallowing problems*. *Increasing mealtime support needs* was also important (*P* = 0.055), and the model included three additional variables that did not reach statistical significance (*P* ≤ 0.05): a quadratic form of *age*, *dementia* and *living in one's own/family home* (see Table 4).

The strongest predictors of *emergency hospitalisation for EDS problems* in our final model were: *increasing mealtime support needs*, followed by *epilepsy*, a need for *full mealtime support* and *physical disability*. The model also included *diagnosed dysphagia*, and a quadratic form of *age* (see Table 5), although neither achieved statistical significance. People with a *physical disability* had (unexpectedly) lower odds of emergency hospitalisations related to EDS than individuals with no disability.

Finally, we should note that the quadratic age relationship, the best fitting according to AIC (Table 3), does not *just* represent the age effect; the age form has the most freedom (compared to the dichotomous binary variables) to model unmeasured confounders and interactions with age – effectively, the age form can account for more of the variation in our outcome (recalling that we considered 13 possible age forms). Hence, we cannot interpret the age terms strictly as age effects, but as a representation of effects correlated with the age form. The quadratic form of age indicates that older and younger individuals have an increased risk of adverse outcomes. This is most likely a proxy for the greater early‐life difficulties faced by some individuals with the most profound forms of intellectual and physical disabilities (and some not surviving to later life), as well as increased morbidity among older individuals.

### Comparison with existing literature

Most studies exploring predictors of respiratory infections have focused on other high‐risk groups (e.g. older adults without ID). Although the association between dysphagia and respiratory infections is fairly consistent in the literature addressing elderly populations, its relative importance in the development of such infections, compared to other risk factors, remains uncertain (Langmore *et al.* 1998; Loeb *et al.* 1999; Langmore *et al.* 2002; Marik & Kaplan 2003). When the relative weight of other factors was considered, Hibberd *et al.* (2013) found that dysphagia was only the sixth strongest predictor of aspiration pneumonia, behind variables indicating tube/oral feeding, mobility, age and feeding dependency, but more important than bedfast, number of comorbidities or medications, gender and other conditions like chronic obstructive pulmonary disease and cerebrovascular accident (i.e. stroke). In our sample, which included young and older adults with ID, we found that having *diagnosed dysphagia* was not a strong predictor of *respiratory infections*. However, individuals with *swallowing problems* had odds of a respiratory infection five times those of their peers with no reported *swallowing problem*s. This could relate to the broader nature of *swallowing problems*, which included most participants (88%, 40/45) with *diagnosed dysphagia* alongside individuals with no such diagnosis. Alternatively, it could indicate that people with *diagnosed dysphagia* have better EDS management strategies in place, helping to prevent *respiratory infections*.

In our study, *epilepsy* strongly predicted both *respiratory infections* and *emergency hospitalisation*. Epilepsy is more prevalent among individuals with ID, generally harder to control in this group, and is associated with more severe ID (Lhatoo & Sander 2001). We did not expect *epilepsy* to be a strong predictor of *EDS‐related hospitalisations* or *respiratory infections*; thus, we only collected information on the presence or absence of epilepsy, rather than more detailed information (e.g. epilepsy severity). More detailed information could be useful for future research. Previous research comparing adults with ID and epilepsy to those without epilepsy indicates that the former have a higher prevalence of multiple skills deficits: for example, even when adjusted for age, sex and level of understanding, adults with epilepsy were three‐and‐a‐half times more likely to have ‘feeding and drinking’ skill deficits (*P* < 0.001) (McGrother *et al.* 2006). These impairments likely contribute to both mealtime support needs and adverse outcomes. Chadwick & Jolliffe (2009) also found that around half (49.5%) of the adults with ID and dysphagia in their study had epilepsy, which they note is higher than the 15–30% expected from previous literature on adults with ID. They suggest that this could be due to the shared (neurological) aetiology of epilepsy and dysphagia, or to the fact that people with epilepsy are at increased risk of asphyxiation and aspiration when eating and drinking after a seizure (Chadwick & Jolliffe 2009). Increased risk of aspiration and asphyxiation post‐seizure may explain why epilepsy is associated with an increased risk of respiratory infections and EDS‐related emergency hospitalisations here. Our results support Chadwick & Jolliffe's (2009) call for additional research addressing complex interactions between epilepsy and dysphagia/other EDS problems.

Among older people, requiring *full support* with feeding is an important known predictor of aspiration pneumonia (Langmore *et al.* 1998; Marik & Kaplan 2003). While requiring *full mealtime support* (being PEG‐fed or fed entirely by someone else) predicted *emergency hospitalisation* in our participants, it was not a strong predictor in the final model for *respiratory infections*. Eyman *et al.* (1993) found that, amongst people with ID, being unable to self‐feed was associated with shorter life expectancy. Additionally, most deaths in their study population were due to respiratory infections, which are more common amongst people with severely limited/no mobility (overlapping with inability to self‐feed), as immobility limits ‘pulmonary ventilation’, which aside from other issues with swallowing or feeding, increases susceptibility to respiratory infections (Eyman *et al.* 1993). However, in our study, having *increasing mealtime support needs* was a stronger risk factor for *respiratory infections* than requiring *full mealtime support*, and the strongest predictor of *emergency hospitalisation*. The significance of *increasing mealtime support needs* as a predictor of both adverse outcomes suggests two possible, although not mutually exclusive, explanations. First, deteriorating EDS skills (and for *hospitalisation*, receiving *full mealtime support*) could predict adverse outcomes simply because these individuals are more likely to experience serious illnesses and complications, regardless of intervention. Alternatively, it could signal that, although an individual's mealtime support may once have been appropriate for their needs, it has not kept pace with changes to their physical/mental health or lifestyle, and is therefore unable to adequately prevent serious adverse outcomes such as respiratory infections and hospitalisations.

Langmore *et al.* (1998) suggested that ‘dependence for feeding’, when considered an indicator of functional capacity, is amongst the later activities to succumb to declining ability and increasing dependence. Thus, our variable for *increasing mealtime support needs* can be interpreted as an indicator of declining skills and increasing dependence. Balandin *et al.*'s (2009) qualitative work with Australian adults with CP, exploring changing mealtime support needs, further illustrates the need for regular monitoring and reassessment of mealtime support among adults with EDS problems. Regular mentoring and reassessment are especially important where people lack the ability to recognise and report changes in their support needs. Providing appropriate support at mealtimes is a complex interpersonal interaction, requiring co‐operative work from both those receiving and providing support (Ball *et al.* 2012, Guthrie & Stansfield 2017), although further research in this area is needed.

With respect to hospitalisation, a US‐based study by Venkat *et al.* (2011) found that being PEG fed and having limited or no mobility were significant predictors of all‐cause emergency department visits amongst adults with ID. Our findings are broadly consistent, as requiring *full mealtime support* (being PEG‐fed or fed entirely by someone else) was a good predictor of *EDS‐related emergency hospitalisation* in our study, although *limited/no mobility* was not. In fact, individuals with a *physical disability* were less likely to experience EDS‐related hospitalisations.

### Strengths and limitations

It is important to note that this research was exploratory and that the prognostic models reported have not been validated. Validation is an important and necessary step in prognostic model development (Altman *et al.* 2009; Moons *et al.* 2009). Ideally, any developed prognostic model is validated on a different sample of the population being considered (Altman *et al*. 2009). A common approach to validation is to randomly split datasets into a ‘training’ and ‘validation’ samples (Altman *et al.* 2009): the training data are used to determine the ‘best’ model, which is then tested on the validation data. Given that we started with a relatively small dataset, this was unfeasible. We have, however, taken steps to explore the robustness of the final models. We have provided a bootstrapped range for AUCs to give an indication of their uncertainty and compared the prognostic models with the linear‐age models and the model averaging across age forms to investigate the robustness of parameters. While these efforts do not remove the need to validate the prognostic models, they do represent some of the best approaches that can be applied in the present situation.

Additionally, ethical restrictions relating to the capacity to consent complicate the recruitment and involvement of adults with ID in primary research (Lennox *et al.* 2005; Iacono & Carling‐Jenkins 2012). Here, only 20% of the individuals invited to participate consented or had favourable advice given for participation, possibly leading to selection bias and therefore limiting the generalisability of findings. Further selection bias may have resulted from the need to seek favourable advice from consultees (under the *Mental Capacity Act 2005*) to enable the participation of adults who lacked capacity. Our results should also be interpreted cautiously, as some of our data collection may be subject to recall bias: some variables relate to events occurring in a fixed reference period, such as the number of respiratory infections during the 12 months prior to interview. Two participants were missing information on dysphagia diagnosis and one on ID severity, as these details were not available, indicating that even seemingly objective diagnoses are sometimes subject to uncertainty. Nevertheless, we have investigated an under‐researched subject in a vulnerable population. Through community‐based primary data collection, we gathered information on a range of issues that would have been impossible to investigate using routinely‐collected data.

### Implications for research and practice

As this analysis is exploratory, we have several recommendations for future research. First, testing these predictive models on another population of men and women with ID and EDS problems should be considered an essential next step. This is a necessary part of accurate model formulation and is needed to validate our findings. Second, as epilepsy was a strong predictor of the EDS‐related adverse outcomes we investigated, we strongly recommend research investigating links between epilepsy, EDS problems and respiratory infections. Finally, our results indicate that the increased risk of negative outcomes is not limited to people with clinically diagnosed dysphagia. In fact, *caregiver‐reported swallowing problems* were a better predictor of *respiratory infections* than *diagnosed dysphagia*. Future studies of EDS problems should therefore seek to include individuals with caregiver‐reported difficulties, alongside those with clinically *diagnosed dysphagia*. Given the increasing risk of negative outcomes as age increases beyond around 50, and that dementia was not a significant predictor, future research could explore if other age‐related conditions are useful predictors.

Although we are unable to provide definitive evidence, our findings suggest that in order to prevent future respiratory infections amongst adults with ID who require support at mealtimes, certain individuals may benefit from closer monitoring. These include individuals with a *history of respiratory infections* resulting in GP contact or hospitalisation, who have *epilepsy* and *difficulty swallowing*, as well as those with declining EDS skills resulting in *increasing mealtime support needs.* Similar strategies might also prevent EDS‐related hospitalisations among adults with *increasing mealtime support needs*, *epilepsy* and those who receive *full mealtime support* for oral or enteral feeding.

*Respiratory infections* and *hospitalisations* have substantial personal costs for individuals with ID, and the carers or paid support workers who help them live independent lives, alongside financial costs for the NHS (Perez *et al*. 2015). Qualitative studies involving individuals with lifelong disability vividly illustrate the impact of feeding changes on mental health and quality of life (Balandin *et al.* 2009), while the Confidential Inquiry into Premature Deaths of People with Learning Disabilities (Heslop *et al.* 2013) definitively showed that people with ID are less likely to receive the healthcare they need.

We would argue for increased attention to EDS problems across a hierarchy of intervention. This begins with family and paid caregivers who provide intimate daily support, often without specialist training. Tools, such as the Nutrition and Swallowing Checklist (Stewart 2003; von Konigsmark *et al.* 2003), can help caregivers identify individuals with changing EDS needs and seek support from GPs. General Practitioners, in turn, should be particularly concerned by repeated respiratory infections. An assessment of basic EDS skills could be added to annual ID health checks (Hoghton 2010), through simple questions regarding mealtime support needs (e.g. ability to feed oneself), whether support needs have increased since the last check, and whether any new or persistent swallowing difficulties have developed. The final opportunity for intervention lies with specialists, e.g. the SLTs and dietitians who can diagnose conditions like dysphagia, and provide tailored guidelines for caregivers. These guidelines – given the centrality of eating and drinking to both physical and psychological wellbeing – should be regularly reviewed so as to ensure that where a person's mealtime support needs change, such changes are not overlooked.

## Funding

This paper presents independent research commissioned by the National Institute for Health Research (NIHR) under its Research for Patient Benefit (RfPB) programme (grant reference number PB‐PG‐0906‐11098). A.P. Wagner, I.C.H. Clare and A.J. Holland are supported by the National Institute for Health Research (NIHR) Collaboration for Leadership in Applied Health Research and Care East of England at Cambridgeshire and Peterborough NHS Foundation Trust. The views expressed are those of the authors and not necessarily those of the NHS, the NIHR or the Department of Health. S. R. White was supported by the Medical Research Council (Unit Programme number U105292687).

## Conflicts of interest

None declared.

## Ethical approval

The project received ethical approval from the NHS Cambridgeshire 3 Research Ethics Committee, UK (REC reference number 07/H0306/98). All participants with capacity gave consent to participate in the study. In accordance with the *Mental Capacity Act (England and Wales) 2005*, we sought advice from consultees regarding the participation of men and women who lacked the capacity to decide whether or not to participate.

## Contributors

M.R., A.J.H. and I.C.H.C. planned and designed the study, obtained funding and supervised the collection of data by S.L.B. S.L.B. was also responsible for the day‐to‐day running of the study and for refining the methodology in light of practical constraints. A.P.W. and C.M.P. were responsible for analysing the data (with statistical advice from S.R.W. and guidance from S.L.B.) and for writing the manuscript. All authors contributed to the editing of the manuscript for which M.R. is guarantor.

## Supporting information

Appendix S1: Additional methodological details regarding the selection of different age models in the statistical analysis.
**Figure S1:** Sensitivity and specificity plot of the best prognostic model using backwards stepwise AIC for respiratory infection within the previous year.
**Figure S2:** Sensitivity and specificity plot of the best prognostic model using backwards stepwise AIC for emergency hospitalisation related to EDS within the previous year.

Data S1. Supporting info item
