# Supplementary material for: Prognostic models for identifying adults with intellectual disabilities and mealtime support needs who are at greatest risk of respiratory infection and emergency hospitalisation
Source: J Intellect Disabil Res. 2017 May 11;61(8):737–54. doi: 10.1111/jir.12376 (PMC5518212; doi:10.1111/jir.12376)
Supplement: Supplementary file 1 — Data S1. Supporting info item [file JIR-61-737-s001.docx]

**SUPPORTING INFORMATION (WEB-ONLY)**

Appendix S1: Additional methodological details regarding the selection of different age models in the statistical analysis

**Modelling age with fractional polynomials**

*Age* is the only continuous variable among the predictors. Royston et al. ([2009](#_ENREF_1), [2006](#_ENREF_3)) suggest that assuming that continuous variables are linear can lead analysts to misinterpret a predictor’s influence on the outcome and therefore to making inaccurate predictions in new populations. It is likely that *age* will not be linear in the present setting, given the possibility of complicated relationships between age and other variables (e.g. age and accommodation: older people with ID may be more likely to like in residential accommodation, as their parents/carers will be older or may have died). There are various approaches to modelling potential non-linearity, including restricted cubic regression splines ([Harrell, 2001](#_ENREF_2)) or fractional polynomials ([Royston and Sauerbrei, 2008](#_ENREF_5), [Sauerbrei and Royston, 1999](#_ENREF_6)). The fractional polynomials approach consists of including a range of various transforms of continuous variables among all the predictors and then applying backward elimination (see Table for a list of the transformations used). Here, we adopted the use of fractional polynomials in the hopes of getting a simple description of the fitted curve and to avoid any anomalous “wiggles” or data/statistical artefacts in our fitted curves that could lead to misinterpretation ([Royston et al., 2009](#_ENREF_4)).

**Exploratory covariate selection for the prognostic models**

Unfortunately, exploratory attempts at applying the fractional polynomials with the standard approach of backward elimination resulted in *all* seven transforms of age being included in prognostic models. This gives an exceedingly complicated relationship with age, resulting in a curve that is not particularly smooth and has many “wiggles”. It is unreasonable to justify such a complex relationship on the basis of the relatively small sample available (n≈130). Therefore, a slightly augmented model fitting approach was adopted, using the two-stage model fitting approach described in the *Methods* section, under Final covariate selection approach for the prognostic models.


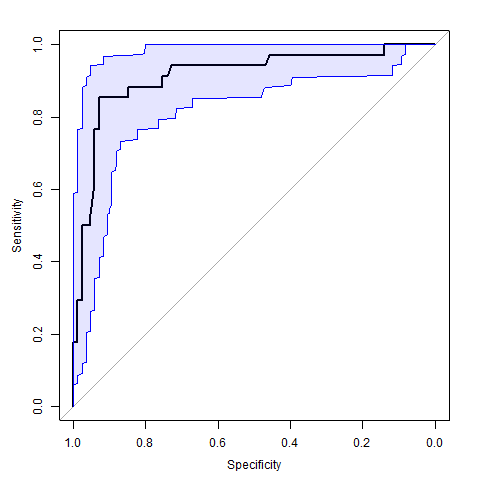


**Figure S1:** Sensitivity and specificity plot of the best prognostic model using backwards stepwise AIC for *respiratory infection* within the previous year.


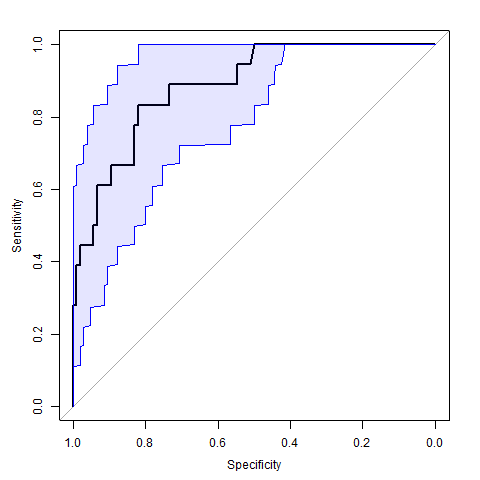


**Figure S2:** Sensitivity and specificity plot of the best prognostic model using backwards stepwise AIC for *emergency hospitalisation related to EDS* within the previous year.

ALTMAN, D. G., VERGOUWE, Y., ROYSTON, P. & MOONS, K. G. M. 2009. Prognosis and prognostic research: validating a prognostic model. *British Medical Journal,* 338.

HARRELL, F. 2001. *Regression Modelling Strategies With Applications to Linear Models, Logistic Regression, and Survival Analysis,* New York, Springer.

ROYSTON, P., ALTMAN, D. G. & SAUERBREI, W. 2006. Dichotomizing continuous predictors in multiple regression: a bad idea. *Statistics in Medicine,* 25**,** 127-141.

ROYSTON, P., MOONS, K. G. M., ALTMAN, D. G. & VERGOUWE, Y. 2009. *Prognosis and prognostic research: Developing a prognostic model*.

ROYSTON, P. & SAUERBREI, W. 2008. *Multivariable Model-building: A Pragmatic Approach to Regression Analysis Based on Fractional Polynomials for Modelling Continuous Variables,* Chichester, John Wiley & Sons.

SAUERBREI, W. & ROYSTON, P. 1999. Building multivariable prognostic and diagnostic models: transformation of the predictors by using fractional polynomials. *Journal of the Royal Statistical Society: Series A (Statistics in Society),* 162**,** 71-94.
